# Supplementary material for: Causality between Sex Hormones and Bone Mineral Density in Childhood: Age- and Tanner-Stage-Matched Sex Hormone Level May Be an Early Indicator of Pediatric Bone Fragility
Source: Biomedicines. 2024 May 25;12(6):1173. doi: 10.3390/biomedicines12061173 (PMC11200954; doi:10.3390/biomedicines12061173)
Supplement: Supplementary file 1 [file biomedicines-12-01173-s001.zip › biomedicines-2978617-supplementary.pdf]

Supplementary Table S1a. Univariate and multivariate regression analyses of factors associated with the lumbar spine bone mineral density for hemato-oncologic diseases.

	LSBMD *Z-scores*							
	Male				Female			
	Univariate		Multivariate		Univariate		Multivariate	
	β	*p*	β	*p*	β	*p*	β	*p*
**Age at diagnosis**	**−0.07**	***<0.001***	**−0.09**	***<0.001***	**−0.05**	***0.006***	**-0.06**	***0.002***
**†BMI**	**0.36**	***<0.001***	**0.39**	***<0.001***	**0.29**	***<0.001***	**0.28**	***<0.001***
**‡LH**	0.27	*0.492*			−0.06	*0.299*		
**‡FSH**	0.06	*0.805*			**−0.13**	***0.018***	−0.06	*0.343*
**‡Testosterone**	**0.87**	***0.003***	**1.33**	***<0.001***				
**‡Estradiol**					**0.14**	***0.024***	0.07	*0.327*

†Indicates sex- and age-matched *Z-scores*. ‡Indicates sex- and Tanner stage-matched *Z-scores*. BMI, body mass index; FSH, follicular-stimulating hormone; LH, luteinizing hormone, LSBMD, lumbar spine bone mineral density.

Supplementary Table S1b. Univariate and multivariate regression analyses of factors associated with the lumbar spine bone mineral density for rheumatoid diseases.

	LSBMD *Z-scores*							
	Male				Female			
	Univariate		Multivariate		Univariate		Multivariate	
	β	*p*	β	*p*	β	*p*	β	*p*
**Age at diagnosis**	0.17	*0.226*			0.07	*0.213*		
**†BMI**	0.25	*0.544*			**0.46**	***0.004***	**0.42**	***0.008***
**‡LH**	**8.40**	***0.049***	**8.40**	***0.049***	−0.14	*0.345*		
**‡FSH**	2.01	*0.482*			−0.79	*0.779*		
**‡Testosterone**	1.63	*0.312*						
**‡Estradiol**					0.34	*0.104*	0.07	*0.327*

†Indicates sex- and age-matched *Z-scores*. ‡Indicates sex- and Tanner stage-matched *Z-scores*. BMI, body mass index; FSH, follicular-stimulating hormone; LH, luteinizing hormone, LSBMD, lumbar spine bone mineral density.

Supplementary Table S1c. Univariate and multivariate regression analyses of factors associated with the lumbar spine bone mineral density for gastrointestinal diseases.

	LSBMD *Z-scores*							
	Male				Female			
	Univariate		Multivariate		Univariate		Multivariate	
	β	*p*	β	*p*	β	*p*	β	*p*
**Age at diagnosis**	-0.31	*0.822*			0.12	*0.057*		
**†BMI**	**0.73**	***0.015***	**0.73**	***0.015***	0.16	*0.257*		
**‡LH**	**5.16**	***0.042***	0.28	*0.273*	0.04	*0.971*		
**‡FSH**	0.14	*0.939*			-2.39	*0.358*		
**‡Testosterone**	1.57	*0.056*						
**‡Estradiol**					0.14	*0.577*		

†Indicates sex- and age-matched *Z-scores*. ‡Indicates sex- and Tanner stage-matched *Z-scores*. BMI, body mass index; FSH, follicular-stimulating hormone; LH, luteinizing hormone, LSBMD, lumbar spine bone mineral density.

Supplementary Table S1d. Univariate and multivariate regression analyses of factors associated with the lumbar spine bone mineral density for endocrinologic diseases.

	LSBMD *Z-scores*							
	**Male**				**Female**			
	**Univariate**		**Multivariate**		**Univariate**		**Multivariate**	
	β	*p*	β	*p*	β	*p*	β	*p*
**Age at diagnosis**	**-0.31**	***0.009***	**-0.31**	***0.009***	**-0.16**	***0.004***	-0.36	*0.125*
**†BMI**	0.45	*0.140*			**0.31**	***0.049***	0.37	*0.057*
**‡LH**	2.83	*0.108*			0.36	*0.350*		
**‡FSH**	1.21	*0.223*			-0.07	*0.623*		
**‡Testosterone**	-0.10	*0.943*						
**‡Estradiol**					**0.65**	***0.006***	**0.65**	***0.006***

†Indicates sex- and age-matched *Z-scores*. ‡Indicates sex- and Tanner stage-matched *Z-scores*. BMI, body mass index; FSH, follicular-stimulating hormone; LH, luteinizing hormone, LSBMD, lumbar spine bone mineral density.

Supplementary Table S2. Univariate and multivariate regression analyses of factors associated with height-adjusted lumbar spine bone mineral density of male and female subjects.

	**Height-adjusted LSBMD *Z-score*s**							
	**Male**				**Female**			
	**Univariate**		**Multivariate**		**Univariate**		**Multivariate**	
	β	*p*	β	*p*	β	*p*	β	*p*
**Age at diagnosis**	**-0.08**	***<0.001***	**-0.073**	***<0.001***	-0.03	*0.084*		
**†LH**	**-1.343**	***0.001***	**-1.157**	***0.005***	-0.069	*0.162*		
**†FSH**	-0.454	*0.065*			**-0.09**	***0.049***	-0.037	*0.521*
**†Testosterone**	**-0.595**	***0.041***	-0.368	*0.195*				
**†Estradiol**					0.107	*0.089*		

†Indicates sex- and Tanner stage-matched *Z-score*s. BMI, body mass index; FSH, follicular-stimulating hormone; LH, luteinizing hormone, LSBMD, lumbar spine bone mineral density.

Supplementary Table S3a. Logistic regression models demonstrating the effect of luteinizing hormone, follicular-stimulating hormone, testosterone, and estradiol on lumbar spine bone mineral density for hemato-oncologic diseases.

	**LSBMD *Z-scores***											
	**>–1.0**				**>–2.0**				**>–3.0**			
	**Male**		**Female**		**Male**		**Female**		**Male**		**Female**	
	**OR**	***p***	**OR**	***p***	**OR**	***p***	**OR**	***p***	**OR**	***p***	**OR**	***p***
**†LH**												
**‡Adjusted**	**19.94**	***0.022***	0.92	*0.476*	2.76	*0.725*	1.96	*0.172*	0.001	*0.258*	2.44	*0.509*
**†FSH**												
**‡Adjusted**	0.59	*0.446*	1.09	*0.495*	5.30	*0.116*	0.76	*0.302*	**840.99**	***0.038***	0.75	*0.680*
**†Testosterone**												
**‡Adjusted**	**5.28**	***0.004***			**54.03**	***<0.001***			**1615.58**	***0.004***		
**†Estradiol**												
**‡Adjusted**			1.13	*0.383*			**2.03**	***0.002***			**19.99**	***0.016***

†Indicates sex- and Tanner stage-matched *Z-scores*. ‡Adjusted for age of diagnosis of underlying disease, and age- and sex-matched body mass index *Z-score*. FSH, follicular-stimulating hormone; LH, luteinizing hormone, LSBMD, lumbar spine bone mineral density; OR, odds ratio.

Supplementary Table S3b. Logistic regression models demonstrating the effect of luteinizing hormone, follicular-stimulating hormone, testosterone, and estradiol on lumbar spine bone mineral density for rheumatoid diseases.

	**LSBMD *Z-scores***											
	**>–1.0**				**>–2.0**				**>–3.0**			
	**Male**		**Female**		**Male**		**Female**		**Male**		**Female**	
	**OR**	***p***	**OR**	***p***	**OR**	***p***	**OR**	***p***	**OR**	***p***	**OR**	***p***
**†LH**												
**‡Adjusted**	8036.63	*0.149*	0.64	*0.325*	40.80	*0.737*	0.55	*0.240*	N/A		N/A	
**†FSH**												
**‡Adjusted**	0.26	*0.696*	0.16	*0.391*	1.57	*0.942*	650.39	*0.406*	N/A		N/A	
**†Testosterone**												
**‡Adjusted**	480.64	*0.186*			18.14	*0.487*			N/A			
**†Estradiol**												
**‡Adjusted**			1.90	*0.391*			2.42	*0.141*			N/A	

†Indicates sex- and Tanner stage-matched *Z-scores*. ‡Adjusted for age of diagnosis of underlying disease, and age- and sex-matched body mass index *Z-score*. FSH, follicular-stimulating hormone; LH, luteinizing hormone, LSBMD, lumbar spine bone mineral density; OR, odds ratio; N/A, non-applicable.

Supplementary Table S3c. Logistic regression models demonstrating the effect of luteinizing hormone, follicular-stimulating hormone, testosterone, and estradiol on lumbar spine bone mineral density for gastrointestinal diseases.

	**LSBMD *Z-scores***											
	**>–1.0**				**>–2.0**				**>–3.0**			
	**Male**		**Female**		**Male**		**Female**		**Male**		**Female**	
	**OR**	***p***	**OR**	***p***	**OR**	***p***	**OR**	***p***	**OR**	***p***	**OR**	***p***
**†LH**												
**‡Adjusted**	N/A		2.09	*0.734*	N/A		0.79	*0.930*	N/A		N/A	
**†FSH**												
**‡Adjusted**	N/A		0.01	*0.328*	N/A		0.01	*0.353*	N/A		N/A	
**†Testosterone**												
**‡Adjusted**	N/A				N/A				N/A			
**†Estradiol**												
**‡Adjusted**			1.23	*0.328*			2.04	*0.466*			N/A	

†Indicates sex- and Tanner stage-matched *Z-scores*. ‡Adjusted for age of diagnosis of underlying disease, and age- and sex-matched body mass index *Z-score*. FSH, follicular-stimulating hormone; LH, luteinizing hormone, LSBMD, lumbar spine bone mineral density; OR, odds ratio; N/A, non-applicable.

Supplementary Table S3d. Logistic regression models demonstrating the effect of luteinizing hormone, follicular-stimulating hormone, testosterone, and estradiol on lumbar spine bone mineral density for endocrinologic diseases.

	LSBMD *Z-scores*											
	>–1.0				>–2.0				>–3.0			
	Male		Female		Male		Female		Male		Female	
	**OR**	***p***	**OR**	***p***	**OR**	***p***	**OR**	***p***	**OR**	***p***	**OR**	***p***
**[†]LH**												
**[‡]Adjusted**	N/A		0.34	*0.739*	N/A		N/A		57.32	*0.774*	N/A	
**[†]FSH**												
**[‡]Adjusted**	N/A		1.25	*0.755*	N/A		N/A		0	*0.437*	N/A	
**[†]Testosterone**												
**[‡]Adjusted**	N/A				N/A				2.05	*0.834*		
**[†]Estradiol**												
**[‡]Adjusted**			4.90	*0.227*			N/A				N/A	

[†]Indicates sex- and Tanner stage-matched *Z-scores*. [‡]Adjusted for age of diagnosis of underlying disease, and age- and sex-matched body mass index *Z-score*. FSH, follicular-stimulating hormone; LH, luteinizing hormone, LSBMD, lumbar spine bone mineral density; OR, odds ratio; N/A, non-applicable.

Supplementary Table S4. Logistic regression models demonstrating the effect of luteinizing hormone, follicular-stimulating hormone, testosterone, and estradiol on height-adjusted lumbar spine bone mineral density.

	**LSBMD *Z-scores***											
	**>−1.0**				**>−2.0**				**>−3.0**			
	**Male**		**Female**		**Male**		**Female**		**Male**		**Female**	
	**OR**	***p***	**OR**	***p***	**OR**	***p***	**OR**	***p***	**OR**	***p***	**OR**	***p***
**†LH**												
**‡Adjusted**	**0.2**	***0.029***	0.92	*0.334*	0.33	*0.546*	0.95	*0.655*	0.01	*0.117*	1.25	*0.697*
**†FSH**												
**‡Adjusted**	**0.46**	***0.03***	0.976	*0.785*	0.68	*0.358*	0.82	*0.106*	0.45	*0.141*	0.8	*0.323*
**†Testosterone**												
**‡Adjusted**	0.53	*0.133*			0.45	*0.16*			0.55	*0.461*		
**†Estradiol**												
**‡Adjusted**			**1.31**	***0.025***			**2.66**	***<0.001***			**10.4**	***0.004***

†Indicates sex- and Tanner stage-matched *Z-scores*. ‡Adjusted for the underlying disease, age of diagnosis of underlying disease. FSH, follicular-stimulating hormone; LH, luteinizing hormone, LSBMD, lumbar spine bone mineral density; OR, odds ratio.
